# Bis[(1*H*-1,2,3-benzotriazol-1-yl)methyl 2,2-dimethyl­propano­ato-κ*N* ^3^]dichlorido­copper(II)

**DOI:** 10.1107/S1600536812015486

**Published:** 2012-04-18

**Authors:** Gang Cao, Ting Guo, Sen Xu

**Affiliations:** a Department of Stomatology, Nanjing Jinling Hospital, Nanjing University School of Medicine, 305 East Zhongshan Road, 210002 Nanjing, Jiangsu Province, People’s Republic of China; b Department of Applied Chemistry, School of Material Science and Engineering, Nanjing University of Aeronautics and Astronautics, Nanjing, Jiangsu Province 210016, People’s Republic of China

## Abstract

In the title compound, [CuCl_2_(C_12_H_15_N_3_O_2_)_2_], the Cu^II^ ion is located on an inversion center and is four-coordinated in a distorted square-planar geometry by two chloride anions and two N atoms from two (1*H*-1,2,3-benzotriazol-1-yl)methyl 2,2-dimethyl­propano­ate ligands. The Cl—Cu—N angles of 90.55 (9) and 89.45 (9)° are close to ideal values. In the crystal, weak π–π stacking inter­actions are observed between inversion-related benzene rings [centroid–centroid distance = 4.0028 (6) Å].

## Related literature

For related structures, see: Wang (2008 ▶); Tang *et al.* (2011 ▶). For the structure of the free benzotriazole ligand, see: Xu & Shen (2012 ▶).
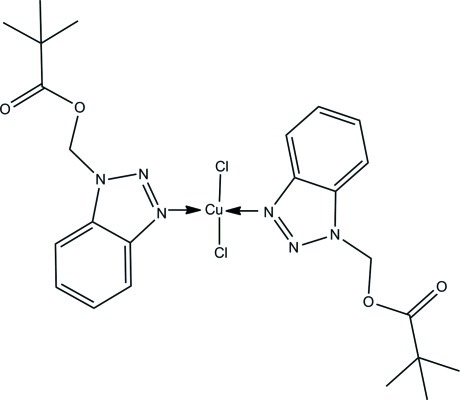


## Experimental

### 

#### Crystal data

[CuCl_2_(C_12_H_15_N_3_O_2_)_2_]
*M* *_r_* = 600.98
Monoclinic, 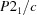

*a* = 10.1929 (19) Å
*b* = 14.576 (2) Å
*c* = 9.2565 (15) Å
β = 96.499 (14)°
*V* = 1366.4 (4) Å^3^
*Z* = 2
Mo *K*α radiation
μ = 1.04 mm^−1^
*T* = 296 K
0.20 × 0.20 × 0.19 mm

#### Data collection

Bruker SMART APEX CCD area-detector diffractometer
Absorption correction: multi-scan (*SADABS*; Sheldrick, 1996 ▶) *T*_min_ = 0.820, *T*_max_ = 0.828
9798 measured reflections
2412 independent reflections
2025 reflections with *I* > 2σ(*I*)
*R*_int_ = 0.048

#### Refinement

*R*[*F* ^2^ > 2σ(*F* ^2^)] = 0.052
*wR*(*F* ^2^) = 0.101
*S* = 1.15
2412 reflections
172 parameters
H-atom parameters constrained
Δρ_max_ = 0.26 e Å^−3^
Δρ_min_ = −0.39 e Å^−3^

### 

Data collection: *SMART* (Bruker, 1998 ▶); cell refinement: *SAINT* (Bruker, 1998 ▶); data reduction: *SAINT*; program(s) used to solve structure: *SHELXS97* (Sheldrick, 2008 ▶); program(s) used to refine structure: *SHELXL97* (Sheldrick, 2008 ▶); molecular graphics: *DIAMOND* (Brandenburg, 1999 ▶); software used to prepare material for publication: *SHELXTL* (Sheldrick, 2008 ▶).

## Supplementary Material

Crystal structure: contains datablock(s) global, I. DOI: 10.1107/S1600536812015486/bh2425sup1.cif

Structure factors: contains datablock(s) I. DOI: 10.1107/S1600536812015486/bh2425Isup2.hkl

Additional supplementary materials:  crystallographic information; 3D view; checkCIF report

## Figures and Tables

**Fig. 1.**
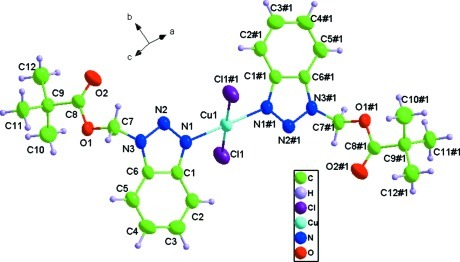
The molecular structure of the title compound with displacement ellipsoids drawn at the 50% probability level (Symmetry code #1: 1-x, -y, -z).

## supplementary crystallographic information

### Comment

We recently synthesized a new benzotriazole derivative, *L*, namely (1*H*-1,2,3-benzotriazol-1-yl)methyl 2,2-dimethylpropanoate (Xu & Shen, 2012). For continuing our work, we now synthesized the title compound, [CuCl_2_(*L*)_2_]. The asymmetric unit contains one half of a complex, with the Cu^II^ ion located on an inversion center (Fig. 1). The metal is four-coordinated in the common square-planar geometry by two chloride anions and two N atoms from two *L* ligands. Bond lengths, Cu—N = 2.018 (3) and Cu—Cl = 2.2377 (11) Å, are comparable to distances found in other reports (Wang, 2008; Tang *et al.*, 2011). In the crystal, there are C—H···O and C—H···Cl weak hydrogen bonds interactions, and weak π–π stacking interactions between inversion-related benzene rings [centroid to centroid distance = 4.0028 (6)°].

### Experimental

(1*H*-benzo[*d*][1,2,3]triazol-1-yl)methyl pivalate (0.25 mmol, Xu & Shen, 2012) and copper chloride (0.25 mmol) were mixed in a round bottom flask, with 10 ml of absolute ethyl alcohol, and stirred for 10 h. After reaction completed, white solids were obtained, which were dissolved in ethanol. Blue crystals suitable for X-ray analysis were obtained by slow evaporation of ethanol, at room temperature.

### Refinement

H atoms were placed in idealized positions and refined using a riding-model approximation, with C—H= 0.93 Å and *U*_iso_(H) = 1.2*U*_eq_(C) for aryl CH, C—H = 0.96 Å and *U*_iso_(H) = 1.5*U*_eq_(C) for methyl CH_3_, C—H = 0.97 Å and *U*_iso_(H) = 1.2*U*_eq_(C) for methylene CH_2_ groups.

### Crystal data

**Table:**

[CuCl_2_(C_12_H_15_N_3_O_2_)_2_]	*F*(000) = 622
*M**_r_* = 600.98	*D*_x_ = 1.461 Mg m^−^^3^
Monoclinic, *P*2_1_/*c*	Mo *K*α radiation, λ = 0.71073 Å
Hall symbol: -P 2ybc	Cell parameters from 3200 reflections
*a* = 10.1929 (19) Å	θ = 2.5–26.6°
*b* = 14.576 (2) Å	µ = 1.04 mm^−^^1^
*c* = 9.2565 (15) Å	*T* = 296 K
β = 96.499 (14)°	Block, blue
*V* = 1366.4 (4) Å^3^	0.20 × 0.20 × 0.19 mm
*Z* = 2	

### Data collection

**Table:**

Bruker SMART APEX CCD area-detector diffractometer	2412 independent reflections
Radiation source: fine-focus sealed tube	2025 reflections with *I* > 2σ(*I*)
Graphite monochromator	*R*_int_ = 0.048
Detector resolution: 10.0 pixels mm^-1^	θ_max_ = 25.0°, θ_min_ = 2.0°
φ and ω scans	*h* = −11→12
Absorption correction: multi-scan (SABADS; Sheldrick, 1996)	*k* = −17→17
*T*_min_ = 0.820, *T*_max_ = 0.828	*l* = −10→11
9798 measured reflections	

### Refinement

**Table:**

Refinement on *F*^2^	Primary atom site location: structure-invariant direct methods
Least-squares matrix: full	Secondary atom site location: difference Fourier map
*R*[*F*^2^ > 2σ(*F*^2^)] = 0.052	Hydrogen site location: inferred from neighbouring sites
*wR*(*F*^2^) = 0.101	H-atom parameters constrained
*S* = 1.15	*w* = 1/[σ^2^(*F*_o_^2^) + (0.0095*P*)^2^ + 1.8659*P*] where *P* = (*F*_o_^2^ + 2*F*_c_^2^)/3
2412 reflections	(Δ/σ)_max_ < 0.001
172 parameters	Δρ_max_ = 0.26 e Å^−^^3^
0 restraints	Δρ_min_ = −0.39 e Å^−^^3^
0 constraints	

### Fractional atomic coordinates and isotropic or equivalent isotropic displacement parameters (Å^2^)

**Table:**

	*x*	*y*	*z*	*U*_iso_*/*U*_eq_	
C1	0.3811 (3)	−0.0246 (3)	0.2886 (4)	0.0350 (8)	
C2	0.3663 (4)	−0.1197 (3)	0.2948 (4)	0.0413 (9)	
H2	0.3859	−0.1575	0.2192	0.050*	
C3	0.3216 (4)	−0.1545 (3)	0.4170 (4)	0.0488 (10)	
H3	0.3111	−0.2176	0.4246	0.059*	
C4	0.2909 (4)	−0.0983 (3)	0.5317 (4)	0.0503 (11)	
H4	0.2610	−0.1253	0.6129	0.060*	
C5	0.3036 (4)	−0.0055 (3)	0.5273 (4)	0.0459 (10)	
H5	0.2829	0.0320	0.6028	0.055*	
C6	0.3496 (3)	0.0301 (3)	0.4023 (4)	0.0350 (8)	
C7	0.3690 (4)	0.2011 (3)	0.4420 (4)	0.0436 (10)	
H7A	0.4024	0.1906	0.5430	0.052*	
H7B	0.4236	0.2477	0.4037	0.052*	
C8	0.2046 (4)	0.3077 (3)	0.3507 (4)	0.0442 (10)	
C9	0.0678 (4)	0.3415 (3)	0.3700 (4)	0.0441 (10)	
C10	−0.0319 (4)	0.2630 (3)	0.3583 (6)	0.0661 (14)	
H10A	−0.0393	0.2379	0.2619	0.099*	
H10B	−0.1165	0.2857	0.3783	0.099*	
H10C	−0.0029	0.2161	0.4274	0.099*	
C11	0.0769 (4)	0.3834 (3)	0.5231 (5)	0.0570 (12)	
H11A	0.1074	0.3378	0.5937	0.085*	
H11B	−0.0088	0.4047	0.5417	0.085*	
H11C	0.1375	0.4340	0.5294	0.085*	
C12	0.0271 (5)	0.4150 (3)	0.2566 (5)	0.0610 (13)	
H12A	0.0880	0.4654	0.2690	0.092*	
H12B	−0.0602	0.4363	0.2683	0.092*	
H12C	0.0279	0.3895	0.1611	0.092*	
Cl1	0.31996 (11)	−0.08447 (8)	−0.06653 (11)	0.0563 (3)	
Cu1	0.5000	0.0000	0.0000	0.03856 (19)	
N1	0.4260 (3)	0.0325 (2)	0.1866 (3)	0.0398 (8)	
N2	0.4237 (3)	0.1178 (2)	0.2304 (3)	0.0414 (8)	
N3	0.3767 (3)	0.1173 (2)	0.3608 (3)	0.0375 (7)	
O1	0.2367 (3)	0.23282 (18)	0.4335 (3)	0.0461 (7)	
O2	0.2812 (3)	0.3406 (2)	0.2781 (4)	0.0846 (12)	

### Atomic displacement parameters (Å^2^)

**Table:**

	*U*^11^	*U*^22^	*U*^33^	*U*^12^	*U*^13^	*U*^23^
C1	0.0313 (19)	0.040 (2)	0.0332 (19)	0.0021 (16)	0.0034 (15)	−0.0014 (16)
C2	0.042 (2)	0.043 (2)	0.038 (2)	0.0008 (18)	0.0036 (17)	−0.0064 (18)
C3	0.048 (2)	0.047 (3)	0.049 (2)	−0.005 (2)	−0.0013 (19)	0.005 (2)
C4	0.052 (3)	0.060 (3)	0.040 (2)	−0.010 (2)	0.0068 (19)	0.007 (2)
C5	0.045 (2)	0.057 (3)	0.037 (2)	0.000 (2)	0.0082 (17)	−0.003 (2)
C6	0.032 (2)	0.041 (2)	0.033 (2)	0.0044 (17)	0.0076 (15)	−0.0002 (17)
C7	0.039 (2)	0.043 (2)	0.049 (2)	0.0064 (18)	0.0088 (18)	−0.0093 (19)
C8	0.053 (3)	0.041 (2)	0.040 (2)	0.001 (2)	0.0164 (19)	0.0003 (19)
C9	0.044 (2)	0.041 (2)	0.049 (2)	0.0072 (19)	0.0108 (18)	0.0018 (19)
C10	0.047 (3)	0.061 (3)	0.092 (4)	0.000 (2)	0.014 (3)	0.004 (3)
C11	0.061 (3)	0.057 (3)	0.056 (3)	0.020 (2)	0.020 (2)	0.000 (2)
C12	0.062 (3)	0.064 (3)	0.057 (3)	0.018 (2)	0.007 (2)	0.009 (2)
Cl1	0.0553 (7)	0.0696 (8)	0.0458 (6)	−0.0151 (6)	0.0141 (5)	−0.0123 (5)
Cu1	0.0461 (4)	0.0381 (4)	0.0336 (4)	0.0010 (3)	0.0136 (3)	−0.0033 (3)
N1	0.0450 (19)	0.0386 (19)	0.0374 (18)	0.0017 (15)	0.0107 (14)	−0.0057 (15)
N2	0.046 (2)	0.041 (2)	0.0382 (18)	0.0028 (15)	0.0132 (15)	−0.0009 (15)
N3	0.0396 (18)	0.0411 (19)	0.0328 (16)	0.0078 (15)	0.0087 (13)	−0.0043 (14)
O1	0.0432 (16)	0.0388 (16)	0.0592 (17)	0.0125 (13)	0.0189 (13)	0.0043 (14)
O2	0.078 (2)	0.085 (3)	0.102 (3)	0.034 (2)	0.054 (2)	0.048 (2)

### Geometric parameters (Å, º)

**Table:**

C1—N1	1.375 (5)	C9—C12	1.524 (5)
C1—C6	1.386 (5)	C9—C10	1.526 (6)
C1—C2	1.396 (5)	C9—C11	1.536 (5)
C2—C3	1.364 (5)	C10—H10A	0.9600
C2—H2	0.9300	C10—H10B	0.9600
C3—C4	1.404 (6)	C10—H10C	0.9600
C3—H3	0.9300	C11—H11A	0.9600
C4—C5	1.360 (6)	C11—H11B	0.9600
C4—H4	0.9300	C11—H11C	0.9600
C5—C6	1.397 (5)	C12—H12A	0.9600
C5—H5	0.9300	C12—H12B	0.9600
C6—N3	1.365 (5)	C12—H12C	0.9600
C7—O1	1.418 (4)	Cl1—Cu1	2.2377 (11)
C7—N3	1.442 (5)	Cu1—N1^i^	2.018 (3)
C7—H7A	0.9700	Cu1—N1	2.018 (3)
C7—H7B	0.9700	Cu1—Cl1^i^	2.2377 (11)
C8—O2	1.188 (5)	N1—N2	1.309 (4)
C8—O1	1.352 (5)	N2—N3	1.347 (4)
C8—C9	1.508 (5)		
			
N1—C1—C6	107.3 (3)	C9—C10—H10A	109.5
N1—C1—C2	132.4 (3)	C9—C10—H10B	109.5
C6—C1—C2	120.3 (4)	H10A—C10—H10B	109.5
C3—C2—C1	116.8 (4)	C9—C10—H10C	109.5
C3—C2—H2	121.6	H10A—C10—H10C	109.5
C1—C2—H2	121.6	H10B—C10—H10C	109.5
C2—C3—C4	122.3 (4)	C9—C11—H11A	109.5
C2—C3—H3	118.8	C9—C11—H11B	109.5
C4—C3—H3	118.8	H11A—C11—H11B	109.5
C5—C4—C3	121.8 (4)	C9—C11—H11C	109.5
C5—C4—H4	119.1	H11A—C11—H11C	109.5
C3—C4—H4	119.1	H11B—C11—H11C	109.5
C4—C5—C6	115.9 (4)	C9—C12—H12A	109.5
C4—C5—H5	122.1	C9—C12—H12B	109.5
C6—C5—H5	122.1	H12A—C12—H12B	109.5
N3—C6—C1	104.5 (3)	C9—C12—H12C	109.5
N3—C6—C5	132.6 (4)	H12A—C12—H12C	109.5
C1—C6—C5	122.9 (4)	H12B—C12—H12C	109.5
O1—C7—N3	110.8 (3)	N1^i^—Cu1—N1	180.0
O1—C7—H7A	109.5	N1^i^—Cu1—Cl1	90.55 (9)
N3—C7—H7A	109.5	N1—Cu1—Cl1	89.45 (9)
O1—C7—H7B	109.5	N1^i^—Cu1—Cl1^i^	89.45 (9)
N3—C7—H7B	109.5	N1—Cu1—Cl1^i^	90.55 (9)
H7A—C7—H7B	108.1	Cl1—Cu1—Cl1^i^	180.0
O2—C8—O1	121.0 (4)	N2—N1—C1	110.1 (3)
O2—C8—C9	127.6 (4)	N2—N1—Cu1	120.7 (2)
O1—C8—C9	111.4 (3)	C1—N1—Cu1	129.1 (3)
C8—C9—C12	109.3 (3)	N1—N2—N3	107.0 (3)
C8—C9—C10	111.4 (3)	N2—N3—C6	111.2 (3)
C12—C9—C10	110.5 (4)	N2—N3—C7	120.5 (3)
C8—C9—C11	106.3 (3)	C6—N3—C7	128.1 (3)
C12—C9—C11	109.8 (3)	C8—O1—C7	117.5 (3)
C10—C9—C11	109.5 (4)		
			
N1—C1—C2—C3	178.1 (4)	C6—C1—N1—Cu1	175.6 (2)
C6—C1—C2—C3	−0.8 (5)	C2—C1—N1—Cu1	−3.4 (6)
C1—C2—C3—C4	0.4 (6)	Cl1—Cu1—N1—N2	−131.5 (3)
C2—C3—C4—C5	0.2 (6)	Cl1^i^—Cu1—N1—N2	48.5 (3)
C3—C4—C5—C6	−0.3 (6)	Cl1—Cu1—N1—C1	53.3 (3)
N1—C1—C6—N3	0.4 (4)	Cl1^i^—Cu1—N1—C1	−126.7 (3)
C2—C1—C6—N3	179.5 (3)	C1—N1—N2—N3	−0.3 (4)
N1—C1—C6—C5	−178.4 (3)	Cu1—N1—N2—N3	−176.4 (2)
C2—C1—C6—C5	0.7 (6)	N1—N2—N3—C6	0.6 (4)
C4—C5—C6—N3	−178.6 (4)	N1—N2—N3—C7	176.6 (3)
C4—C5—C6—C1	−0.1 (6)	C1—C6—N3—N2	−0.6 (4)
O2—C8—C9—C12	12.0 (6)	C5—C6—N3—N2	178.0 (4)
O1—C8—C9—C12	−170.4 (3)	C1—C6—N3—C7	−176.2 (3)
O2—C8—C9—C10	134.4 (5)	C5—C6—N3—C7	2.5 (7)
O1—C8—C9—C10	−48.0 (5)	O1—C7—N3—N2	104.3 (4)
O2—C8—C9—C11	−106.5 (5)	O1—C7—N3—C6	−80.5 (5)
O1—C8—C9—C11	71.2 (4)	O2—C8—O1—C7	6.4 (6)
C6—C1—N1—N2	0.0 (4)	C9—C8—O1—C7	−171.4 (3)
C2—C1—N1—N2	−179.0 (4)	N3—C7—O1—C8	−107.4 (4)

Symmetry code: (i) −*x*+1, −*y*, −*z*.
